# Associations between Gender, Alcohol Use and Negative Consequences among Korean College Students: A National Study

**DOI:** 10.3390/ijerph17145192

**Published:** 2020-07-18

**Authors:** Patrick Allen Rose, Hugh Erik Schuckman, Sarah Soyeon Oh, Eun-Cheol Park

**Affiliations:** 1 Department of Technology and Society, State University of New York-Korea, 119 Songdo Moonhwa-Ro, Incheon 21985, Korea; 2 Department of Writing and Rhetoric, University of Utah Asia Campus; 119 Songdo Moonhwa-Ro Yeongsu-Gu, Incheon 21985, Korea; hugh.schuckman@utah.edu; 3 Institute of Health Services Research, Yonsei University, 50 Yonsei-ro, Seodaemun-gu, Seoul 03722, Korea; sarahoh@yuhs.ac (S.S.O.); ecpark@yuhs.ac (E.-C.P.)

**Keywords:** alcohol use, drinking, negative consequences, survey, college students, Korea, national, gender

## Abstract

This study examines Korean college students’ rates and the severity of various negative consequences resulting from the frequency and quantity of alcohol consumption and the unique factors that are affecting this problem in the Korean context in comparison to other countries. It assesses how much gender, age and other associated respondent characteristics mediate alcohol use and the resulting negative consequences among the population. A stratified representative sample of 4803 valid student respondents attending 82 colleges participated in the alcohol consumption survey, of which 95% reported drinking in past 12 months. Drinking is measured by the Alcohol Use Disorders Identification Test-Consumption (AUDIT-C) screening tool. Based on this test, composite scores for each participant were computed and students were grouped into four risk groups: (a) nondrinkers, (b) light drinkers, (c) moderate drinkers and (d) heavy drinkers. Outcome measures include 21 validated items evaluating self-reported alcohol-related negative consequences. Rates of negative consequences are reported for each drinking risk group stratified by gender. Descriptive statistics, stepwise regression, multivariate linear regression and MANOVA tests were used to analyze the data. The study found that female respondents in the sample who consumed alcohol in the past 12 months drank 11.5 percent less than males (AUDIT-C score μ = 6.0 and 6.7, respectively), and there was a greater proportion of females (5.1 percent) who were nondrinkers than males (4.6 percent). Yet, when females drank, they experienced 11.8 percent more negative consequences on average than males (μ = 1.9 and 1.7, respectively). The study attempts to explain this apparent contradiction. The self-reported rates for many individual negative consequences also varied discernibly by gender. The study concludes with suggestions for how alcohol prevention on Korean college campuses would benefit from targeting females and males differently.

## 1. Introduction

It is well known that heavy and frequent drinking is linked to many negative consequences for college students, such as missing class, physical injuries, sexual harassment, conflict with friends, property damage, unplanned sexual intercourse, memory loss and drunk driving among others [1]. However, the evidence specifically on Korea regarding the key factors influencing the prevalence of college student drinking and related negative consequences have been scant. This study helps to address the lack of information about Korean college students’ alcohol use and drinking problems.

Prior to this study, the most recent national survey of Korean college student drinking and the related negative consequences was conducted in 2003, and it was found that Korean students were more likely to drink heavily compared to American students and reported higher incidents of some alcohol-related negative consequences [2]. The only other published research targeting Korean college students highlighted how stress, depression and suicide risk were correlated with excessive alcohol consumption [3,4,5]. A recent article examined whether school policies and education programs play a role in discouraging student drinking behavior in Korea [6].

In contrast to the limited research specifically on college student drinking, numerous studies have documented the effects of alcohol consumption on Korean society as whole. According to data released by Euromonitor in 2014, Korea has the highest per capita hard alcohol consumption rate in the world; consuming an average of 13.7 shots of liquor per week [7]. A 2016 survey by the Ministry of Food and Drug Safety study found that 58 percent of Koreans engaged in high-risk drinking and the rate was even higher among those in their 20s—65 percent [8]. In addition, ongoing longitudinal data on adolescent alcohol use are collected by the Korean Youth Risk Behavior Survey (KYRBS).

There are also many studies that indicate the health, social and economic burdens of high-risk drinking in different ways for Korea [9,10,11]. Some studies demonstrate that Koreans who identify as heavy drinkers during college or earlier were at higher risk of premature cardiovascular disease [12], liver disease [13] and gastrointestinal disease [14]. A cross-sectional study of Korean violent crimes from 2007 to 2009, reveals that about one third of all homicides, violent assaults and sexual assaults were alcohol related [15]. On the road, drunk driving was involved in 9 percent of all traffic accidents and 10 percent of all traffic fatalities in Korea [16].

The overall data indicate that alcohol abuse is a serious public health issue in Korea. Although alcohol misuse can begin prior to students entering college, the transition to college is a critical time when students at a young age develop alcohol-related habits [17]. One study estimates that about half of problem drinkers pickup their alcohol consumption behaviors in college [18]. Considering that about 98 percent of Koreans aged 25–34 graduate from college [19], this time is an especially important transition point for targeting interventions that may have a wide scale impact for Korean society.

### Methodological Background

Drawing on internationally validated instruments, this study focuses on identifying respondent characteristics, including sociodemographic factors that discernably affect the negative consequences stemming from heavy and frequent drinking. To measure alcohol use, this study employed the World Health Organization’s (WHO) Alcohol Use Disorders Identification Test-Consumption (AUDIT-C) brief screening tool. The AUDIT-C test is an extensively validated method for assessing college students’ alcohol consumption, but it has produced different results depending on the context. A 2013 study evaluating the sensitivity of the AUDIT-C tool with Korean college students at Chungnam National University found that it had a 95 to 97 percent positive predictive value for high-risk drinking with a cut-off point of 8 or more [20]. In the Chungnam study, the mean AUDIT-C score for female students was 5.1 and for male students it was 6.7; 42 percent of females and 32 percent of males were identified by the AUDIT-C as high-risk drinkers. These results are consistent with studies of college students in other countries where alcohol consumption is also highly prevalent, as in Korea [21,22,23].

Survey items from the Harvard School of Public Health College Alcohol Study (HCAS) were adopted for this study to track negative consequences. In the United States, the HCAS has surveyed over 50,000 students at 120 colleges to better understand the factors that play a role in producing heavy drinking and the extent of negative consequences experienced by college students [24,25]. A summary of over 80 publications based on HCAS concluded that the risk of negative consequences is greatest at the highest levels of alcohol consumption and most of the alcohol-related problems colleges students experienced occurred among heavy and frequent drinkers [25]. Among HCAS respondents, half of all heavy and frequent drinkers reported experiencing five or more different negative consequences in a two-week period [25].

Based on numerous studies, several contextual factors are known to predict AUDIT-C test results and HCAS defined negative consequences for college students. Heavy and frequent drinking and resulting negative consequences vary greatly among different groups of students within colleges and in different settings. For example, local laws, university policies, access to low-cost alcohol, attitudes about drinking and current drinking rates within a community can promote or discourage drinking [25]. Repeated HCAS studies have found that females and older college students report drinking less than male and younger students, while students living alone off campus drank more than students who lived on campus in supervised environments [25].

The purpose of this study is to determine the effect of Korean college student alcohol consumption levels on resulting negative consequences and discuss the implications of the findings for prevention efforts. It is expected that the general effects of drinking for all students will differ from its effects for certain subgroups. In this regard, this study focuses on identifying the key respondent characteristics that are associated with heavy and frequent drinking among college students in Korea and how they may be unique to the context. The prior research summarized above suggests that female students will report drinking substantially less than male students and will show different patterns of experiencing negative consequences [26,27].

## 2. Methods

In 2017, the Korean Educational Development Institute (KEDI) conducted 5024 in-person surveys with students from Korean four-year universities and liberal arts institutions with a total inferential population of 1,951,940 Korean college students. Data were collected on Korean college campuses by trained interviewers and surveys were administered face-to-face. The lengthy survey took most participants over an hour to complete. To increase response rate, respondents were rewarded with an incentive payment of ten thousand Korean won, or about 9 US dollars. The survey produced 5024 complete student responses out of 7278 approached to participate in the study, producing a response rate of 69 percent. Some surveys needed to be excluded based on incomplete responses, bringing the validated sample size to 4803. The survey design was approved-the Institutional Review Board of Yonsei University College of Medicine granted approval for this survey instrument (Approval Number: Y−2017–0084). See the first publication from this study for more details about the methodology [6].

The KEDI website, which serves as a clearinghouse for Korean education institution information, was the source for all college level data on student, faculty and staff populations. These data stem from a 2017 survey conducted among an inferential population of 1,951,940 university students attending four-year and liberal arts institutions in Korea, and it was used to cross-validate this study’s sample. In other words, the characteristics of colleges and college students included in the study sample were compared to the target sample to confirm it was representative.

The survey defined a standard drink as about 8 g of pure alcohol, to help students make more accurate estimates of their alcohol consumption. Equivalencies of a “standard drink” were included as references at the beginning of the survey, educating respondents of equivalent drink standards according to the Korean Centers for Disease Control and Prevention (KCDC). For the study, a standard drink was equivalent to 1/2 glass of wine, 1 shot of herbal liquor, 2/3 can of beer, 1 glass of draft beer, 1 shot of soju (a common Korean hard liquor), 1 shot of fruit wine or 1 shot of cheonju (refined rice wine).

The survey instrument consisted of questions on drinking habits, alcohol-related negative consequences, social norms, campus drinking policies, participant’s socio-demographics and much more. Specific items were validated by previous college alcohol surveys, such as the Harvard School of Public Health College Alcohol Study (HCAS) [25], the World Health Organization’s (WHO) AUDIT-C screening tool [28], the Korea National Health and Nutrition Examination Survey (KNHANES) [29], and the Korea Youth Risk Behavior Web-based Survey (KYRBS) [30]. In addition to collecting standard demographic data on university students, such as class cohort (year of study), major, GPA, age and gender, the survey queried respondents on other background identifiers, such as involvement in school clubs, spending money, smoking, high school drinking and general health. Information on the psychological well-being of participants was also collected, including stress level, depressive thoughts, and suicidal thoughts.

### 2.1. Measures

Respondent Characteristic Variables: Among the 4803 valid records, the median age of the study participants was 21 years (ranging 18–60 years), and 2447 (51 percent) were females and 2356 (49 percent) were males. Respondent characteristics were grouped into three categories, listed below. Respondents were relatively evenly distributed by college enrollment (university size), gender and year of study (cohort) due to the stratified survey sampling design.

1. College Area-Level: College type, region and student enrollment.
2. Sociodemographic Characteristics: Gender, age, year of study, major, GPA, residence, student club participation and monthly spending.
3. Associated Health Indicators: Smoking status, current smoker, stress level, depressive thoughts, suicidal thoughts and general health.

Alcohol Consumption Variables: There are different well-established international screening tools for assessing alcohol use among any given population [30,31,32,33,34,35,36,37]. Generally, the drinking construct in alcohol consumption surveys is measured in terms of a combination of frequency and quantity of alcohol consumption. The major predictor variable selected for this study was the Alcohol Use Disorders Identification Test-Consumption (AUDIT-C), developed by the World Health Organization (WHO) [28]. The AUDIT-C is an abbreviated initial part of the whole AUDIT screening tool that asks respondents: (a) how often they drink by month and week; (b) how many drinks they have on average during a typical day; (c) how often they drink six or more drinks in one occasion. The AUDIT-C was only administered to respondents who responded affirmatively that they had consumed alcohol at least one time in the past 12 months.

The each of the three AUDIT-C questions has a set of five response options to choose from on a scale of 0 to 4. All the recorded responses were then summed to create a composite (or total) score on a range form 0–12. In this way, the categorical data about drinking recorded by the AUDIT-C items were transformed into a continuous variable. The highest AUDIT-C score in the sample was 11 out of 12, which was reported by 436 respondents.

Generally, higher AUDIT-C scores indicate greater likelihood of problem drinking. A score of 0 reflects no alcohol consumption; 3 or more for women and 4 or more for men is positive; 8 or more is an indication of dependency symptoms and harmful alcohol use [29,36]. Based on this guidance, cutoffs were used to convert AUDIT-C composite scores into four mutually exclusive analysis groups that classify survey participants into four risk categories by their frequency and quantity of alcohol consumption based on standardized drink sizes: (a) 0 as “non-drinkers” at no-risk; (b) 1–4 as “light drinkers” at low-risk; (c) 5–8 as “moderate drinkers” at increased-risk; (d) 9–12 as “heavy and frequent drinkers” at severe-risk [30].

Negative Consequences Variables: The prevalence of negative consequences from drinking is the major dependent variable for this study. Many instruments have been developed to measure alcohol-related problems from clinical [19,32,33,34,35,37] and non-clinical [38,39,40,41,42,43,44,45,46,47,48] perspectives. Both clinical and non-clinical measures of negative consequences have been adopted for this study. The first seven clinical questions are based on a second subset of items from the earlier mentioned AUDIT-C screening tool, which have also been defined by previous research as alcohol dependence symptoms [29,47]. The next 14 negative consequence items are from the Harvard School of Public Health College Alcohol Study (HCAS) and are frequently used in college alcohol surveys [49].

These items are scored from 0–4, depending on the frequency students reported experiencing them, ranging from 0 (never experienced) to 4 (either daily experience for AUDIT questions or four times a month for HCAS questions). To create a composite score for negative consequences, these items were recoded into new dichotomous variables, as either experienced or not (1 or 0, respectively). The total of these 21 dichotomously coded items was recoded into a single new composite score representing the count of negative experiences experienced by participants ranging from 0 to 21. This scoring method converts the categorical data collected by negative consequences items into a continuous scale.

A review of prior research suggests cut-off points of 3 (AUDIT-C) and 5 (HCAS) for the negative consequences composite score (about half for each set), defining “problem” or “high-consequence” drinking [48,49,50,51,52]. For ease of interpretation, the results of the negative consequences composted score were summarized by means and four ranges: 0; 1–2; 3–4; 5 or more. The most extreme value of total negative consequences (7 out of 21) was reported by 56 participants (0.01 percent), and 1724 (35.9 percent) of drinkers reported zero negative consequences.

### 2.2. Analysis

Data were entered into Statistical Package for Social Science Version 26 (SPSS) (IBM Corp., Armonk, NY, USA). The first step of data analysis was summarizing categorical data on gender, alcohol consumption (AUDIT-C composite score) and alcohol-related negative consequences (21 questions taken from the AUDIT-C and HCAS instruments) through descriptive statistics. AUDIT-C composite scores measuring respondents’ drinking rates were reduced into four categorical variables representing different types of drinkers and associated risk: “non-drinkers” at no-risk; “light drinkers” at low-risk; “moderate drinkers” at increased-risk; “heavy and frequent drinkers” at severe-risk [28].

The second step of data analysis was to perform multivariate linear regression tests with the stepwise method to determine which of the many independent respondent characteristics variable (categorical) were statistically significant predictors of the dependent AUDIT-C composite score (continuous) and overall negative consequences (continuous) and to evaluate the strength of the relationships.

The third step of data analysis was to (a) assess the effects of the independent gender (dichotomous) variable on dependent variable of overall alcohol consumption (continuous, but represented descriptively by the different risk groups of light, moderate and heavy drinkers) and (b) the combined effects of gender and alcohol consumption on total negative consequences (continuous, but represented descriptively by the ranges of 0, 1–2, 3–4 and 5 or more). Multivariate linear regression tests were performed with gender, AUDIT-C composite scores and total negative consequences variables.

**Figure 1:**
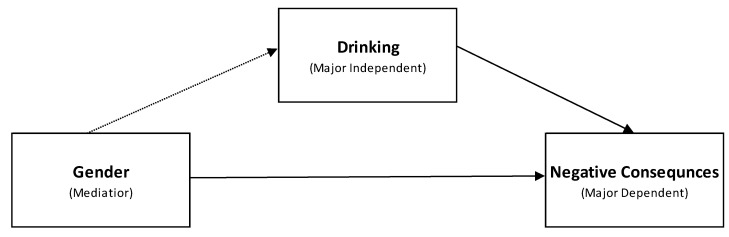
Conceptual Model of Mediated Effects of Alcohol Use and Negative Consequences.

The fourth and final step of data analysis was to determine if males and females experienced 21 specific negative consequences (recoded as dichotomous) at statistically significant different levels (*p* < 0.05). MANOVA (Multivariate Analysis of Variance) tests were performed to allow for the analysis of multiple categorical dependent variables. Figure 1 illustrates the conceptual model for the data analysis strategy.

## 3. Results

### 3.1. Associations between Respondent Characteristics Variables, Alcohol Consumption and Overall Negative Consequences

The first objective of this study is to determine if gender was statistically significantly correlated with drinking and subsequent negative consequences and where gender ranks among all the possible respondent characteristic variables by effect size (R**^2^**). Numerous prior studies provide evidence that underlying social, health, economic and other background characteristics are associated with alcohol-related negative consequences both directly and indirectly through their effects on drinking levels [4,53].

**Figure 2:**
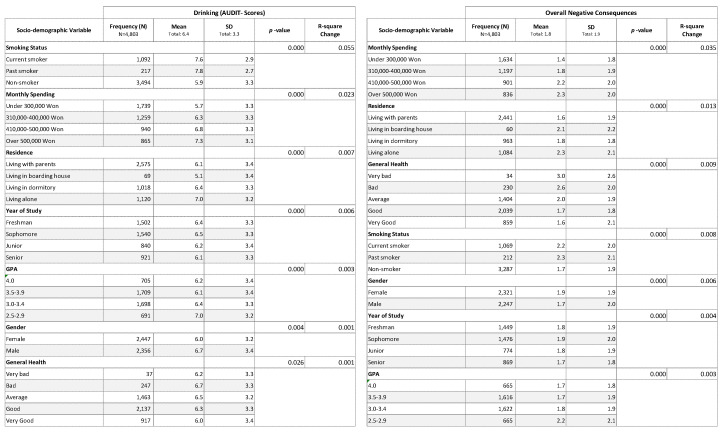
Results of Stepwise Regression Analysis of Alcohol Consumption and Negative Consequences (Overall) for Respondent Characteristics.

Most respondents (4568; 95.1 percent) reported drinking alcohol at least once in the last 12 months. Figure 2 shows that the 7 out of 16 possible respondent characteristics variables included in the analysis were found have significantly statistically (*p* < 0.05) contributed to both regression models for predicting (a) alcohol consumption (AUCIT-C composite scores) and (b) overall negative consequences. These variables included smoking status, monthly spending, residency on campus, year of study, GPA, gender and general health. Gender significantly statistically contributed to the prediction regression models for both drinking (*p* = 0.004) and negative consequences (*p* < 0.000); however, the magnitude of the effect was relatively small (R^2^ = 0.001 and 0.006, respectively).

### 3.2. Associations between Gender, Alcohol Consumption and Overall Negative Consequences

**Figure 3:**
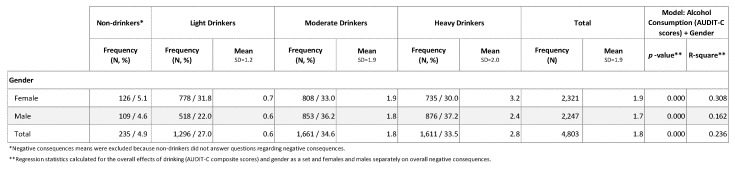
Results of Multivariate Regression Analysis of Negative Consequences (Overall) for Alcohol Consumption Risk Groups.

The second objective of this study was to explore the differences in patterns of drinking and associated negative consequences among women and men in the sample. Numerous previous studies have shown that women tend to report drinking less than men [22,25,54,55,56], and females and males have been shown to experience negative consequences at difference levels. AUDIT-C scores for females were 11.5 percent lower than males (μ = 6.0 and 6.7, respectively); however, Figure 3 shows that females who drank in the past 12 months reported 11.8 percent more negative consequences than males (μ = 1.9 and 1.7, respectively). The difference was even more pronounced among females and males in the “heavy drinkers” risk group (μ = 3.2 and 2.4, respectively). This result indicates that drinking was a stronger predictor of negative consequences for females than males. To confirm this hypothesis, the total sample was split by gender and the regression analysis was performed again. The results found that, for females in the sample, drinking explained 30.8 percent of the variance in overall negative consequences compared to males, where it accounted for 16.2 percent (*p* < 0.000, R^2^ = 0.308 and *p* < 0.000, R^2^ = 0.162, respectively).

**Figure 4:**
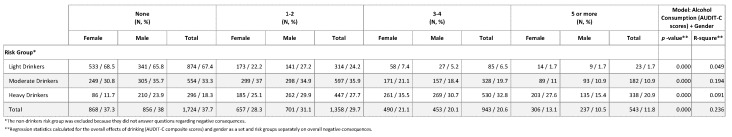
Results of Multivariate Regression Analysis of Negative Consequences (Ranges) Stratified by Gender for Alcohol Consumption Risk Groups.

Figure 4 expounds on the earlier findings by reporting the differences in frequency distribution of negative consequences by ranges (none, 1–2, 3–4 and 5 or more) for each risk group, stratified by gender. On average, the rates of negative consequences are lowest among light drinkers (1296, μ = 0.6) and highest among heavy drinkers (1611, μ = 2.8). The prevalence of heavy drinkers who experienced 5 or more negative consequences was nearly double that of moderate drinkers (20.9 percent vs. 10.9 percent, respectively). It is important to point out that 1724 (35.9 percent) of students who drank in the previous 12 months reported zero negative consequences. Even among heavy drinkers, the largest proportion of participants (296; 18.4 percent) had no negative consequences. Looking at the difference between males and females, 27.6 of female heavy drinkers reported 5 or more negative consequences on average compared to 15.4 percent of males in the same group-a 79.2 percent change. 

### 3.3. Associations between Gender, Alcohol Consumption on Specific Negative Consequences

**Figure 5:**
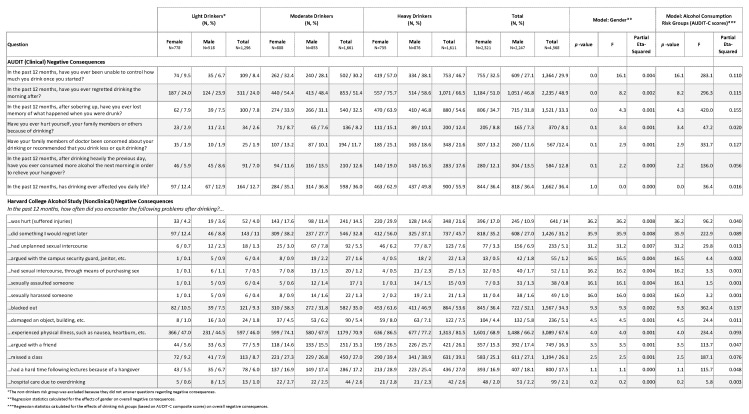
Results of MANOVA of Negative Consequences (Specific) for Alcohol Consumption Risk Groups Stratified by Gender.

The third objective of this study was to determine the effects of gender and drinking on specific alcohol-related negative consequences. One-way ANOVA was conducted to evaluate differences between alcohol consumption risk groups and between women and men across 21 specific negative consequences. Figure 5 shows the frequency distributions of the risk groups stratified by gender for each negative consequence. Risk groups revealed statistically significant (*p* < 0.05) differences across all the dependent variables and between the light and heavy drinkers with one exception—the sexual assault negative consequence.

Figure 5 shows that among all 21 negative consequences variables, the most common were: experienced physical illness (67.6 percent); regretted drinking the morning after (48.9 percent); drinking affected daily life (36.4 percent); blacked out (34.3 percent); memory lost (33.3 percent); doing something that was regretted later (31.2 percent). The variables that were most effected by alcohol consumption were: lost memory (*p* < 0.000, Eta^2^ = 0.155); blacking out (*p* < 0.000, Eta^2^ = 0.137); others recommend drinking less (*p* < 0.000, Eta^2^ = 0.127); inability to control drinking (*p* < 0.000, Eta^2^ = 0.110).

Eight negative consequences did not differentiate between males and females, and the effects of gender on the other 13 were relatively small (Eta^2^ < 0.008). The specific self-reported negative consequences variables, which were shown to be the most different between male and female drinkers were: was hurt (*p* < 0.000, Eta^2^ = 0.008); did something that was regretted later (*p* < 0.000, Eta^2^ = 0.008); had unplanned sexual intercourse (*p* < 0.000, Eta^2^ = 0.007).

## 4. Discussion

This study highlights the effects of gender and other respondent characteristics variables on alcohol consumption (assessed by three AUDIT-C questions on the frequency and quantity of drinking) and resulting negative consequences (adopted from AUDIT-C and NCHS survey instruments). The investigation reveals that most Korean young adults will be exposed to frequent and heavy drinking behaviors in college during this important juncture in their lives. About 95% of survey respondents consumed alcohol in the past 12 months and 34 percent had AUDIT-C composite scores which placed them squarely in the heavy drinker risk group. This result is consistent with previous surveys of alcohol consumption among Korean college students and of college students in other countries where drinking is the norm, such as the United States [25].

As predicted, Korean college students composing the heavy drinkers risk group were found to be at the highest risk for negative consequences. Heavy drinkers reported experiencing an average 2.8 out of 21 negative consequences in the past 12 months, compared to 1.8 among moderate drinkers and 0.6 among light drinkers. This pattern underlines the findings of previous studies in Korea and internationally, which show that incidents of negative consequences are greatest at the highest levels of alcohol consumption among college students [25].

In this study, gender was a statistically significant but a relatively weak predictor of alcohol consumption and negative consequences. Other respondent characteristics, such as living alone and greater monthly spending, were considerably stronger predictors of higher levels of drinking and related problems. However, when the results for females and males were examined independently, it was revealed that while females reported drinking less heavily and frequently on average than males. They also reported higher incidents of negative consequences when they did drink (*p* < 0.000, R^2^ = 0.308 and *p* < 0.000, R^2^ = 0.162, respectively). Females in the sample drank 11.5 percent less than males (AUDIT-C score μ = 6.0 and 6.7, respectively), but reported 11.8 percent more negative consequences (μ = 1.9 and 1.7, respectively).

The finding that females drink less but are at higher risk for alcohol-related problems than males when they do drink is an unexpected contradiction. However, previous studies have also confirmed that females are more at-risk for alcohol-related negative consequences at lower drinking levels than males. Some scholars hypothesize that this situation is due to universal gender differences in alcohol sensitivity [46]. It is believed that the slower metabolism of females, compared to males, increases the effects of alcohol, such as impaired judgment and coordination at lower intake levels in females than males [23,57]. However, other studies point out that alcohol consumption varies greatly in different cultures and biology cannot adequately explain these differences [55,58,59]. Another explanation may be that females tend to be younger than male students, especially in Korea, as Korean males have mandatory military service to complete, which typically delays their graduation. In other words, the maturing effect of age may have confounded the differences between genders [60]. However, this study found that age difference was not a statistically significant predictor of drinking levels even though year in school was somewhat important.

As noted in the introduction to this article, university studies highlight gender differences in the reporting of certain types of negative consequences. Particularly, several previous studies have underscored feelings of regret or guilt after drinking as major differentiating factors between females and males [26,55]. In this study, regret was the second most reported negative consequence overall and females were more likely to report experiencing it. The difference was especially marked within the heavy-drinker risk group. In total, 76 percent of females and 59 percent of males regretted drinking the morning after and, likewise, 56 percent of females and 37 males reported they did something they regretted later. One possible explanation given for this interesting difference is that females are more inclined to internalize problems and use alcohol to relieve their stress or depression, whereas males, in contrast, are more likely to externalize these kinds of negative feelings outwardly as antisocial behaviors [61,62,63,64].

Results for other negative consequences may demonstrate how females and males experience regret for different reasons. Females reported more incidents of rare negative consequence, such as sexual assault and sexual harassment, compared to males who reported higher rates of other uncommon alcohol-related harms, such as unplanned sexual intercourse, purchasing sex and sexually assaulting someone. This conclusion is supported by previous studies, which also report that females are more likely to be the targets of sexual harassment and physical abuse while males are more prone to commit these harms when they drink [27,65,66,67,68].

Another hypothesis is that females report more negative consequences overall compared to males because they feel more regret due to different conceptions of gender roles regarding drinking norms, viewing excessive drinking as more wrong than men [69]. In Korea and many other cultures around the world, heavy drinking is an expression of masculinity for men [70,71,72,73,74,75,76] and, in contrast, abstaining from drinking is linked to the violation of other common feminine norms, such as modesty and sexual fidelity for women [77,78,79]. The pattern of increased feelings of regret after drinking and the overall higher reporting of negative consequences may be a result of societal pressure on female college students to conform to women’s traditional gender roles in society [73,74,80,81]. On the other hand, male student drinkers may boast about their drinking and under-report negative consequences because Korean society greatly encourages masculine risk-taking drinking and minimizes its negative outcomes [63,82].

## 5. Limitations

A general limitation of cross-sectional studies is that, without longitudinal data, causality cannot be ascertained. In this one-off cross-sectional study, it is not possible to unravel factors such as age from an ongoing risk factor, such as heavy drinking, which changes over time. Furthermore, the association between factors may be the result of unobserved setting vulnerability or situation effects on behavior. Although the study had a 69 percent response rate, the sample consisted of volunteers who participated in the research when invited and may not fully represent the diversity of Korean students attending all universities in the county. It is possible that students who agreed to complete the survey are different from those who declined, considering the sensitive topic. Lastly, the self-report method used to collect data on drinking and negative consequences asked participants to recall past experiences as far back as 12 months previously. Asking participants to recall information dependent on their past experiences might introduce a potential bias, as how they remember information on their drinking behaviors may depend on the outcomes of their exposure [83].

## 6. Conclusions

The findings of this study indicate the importance of interventions aimed at addressing heavy and frequent drinking on Korean college campuses. Problem drinking during college is recognized to be a more severe problem in Korea compared to other countries, such as the United States. Yet, the consensus is that Korean universities are still in the early stages of developing systematic prevention programs for the most part. The availability of prevention programs and treatment services related to alcohol use is lower than those related to other social problems, but the primary significance of this study is that it will help to draw attention to the issue of college substance abuse in Korea, which has gone largely unstudied.

Due to insufficient cases, it is difficult to identify which types of prevention programs are promising for Korean colleges. Even though the information is scarce, it can be reasonably assumed that educational programs and campus alcohol policies are usually marginally effective at mitigating high-risk drinking behaviors among Korea college students, unless they are particularly targeted to certain groups [3]. This study emphasizes that the most important group to target is heavy and frequent drinkers who are the most likely to experience alcohol-related negative consequences to a greater extent. These students who regularly engage in high-risk drinking would benefit from alcohol education and treatment and can learn to drink responsibly [3].

Prevention programs in Korea would also benefit from targeting females and males differently. The results of this study indicate that young females are more susceptible to negative consequences resulting from drinking compared to male students who tended to consume alcohol more often and in higher quantities. Prevention programs should recognize that female college students are more vulnerable to certain rare negative consequences, such as unplanned sex, sexual harassment and sexual assault. Female students would benefit from learning about protective and harm-reduction tactics while drinking to reduce risks associated with alcohol consumption and later regrets. On the other hand, male students reported higher incidents of different rare negative consequences involving outwardly aggressive and sometimes violent behavior. Male students could use education and counseling focusing on helping them to learn to drink less destructively and respect their female counterparts. Furthermore, Korean colleges should consider engaging male students in grassroots violence prevention activities aimed at re-defining masculinity in the context of campus culture.

Gender difference is important; however, this study briefly noted that other respondent characteristics appear to be me more influential, such as “living situation” and “available spending money”. Furthermore, supplementary community-based research is needed to identify the differential cognitive and social factors that may amplify social risk factors, such as gender role norms, stereotypes, peer modeling, alcohol expectancies, interpersonal relationship skills and drinking motives, among others. Future reports based on the dataset will focus on these topics. To be more effective, it is recommended that college alcohol educational-based prevention programs in Korea incorporate strategies to address underlying environmental and cultural causes [84]. Korea would benefit from experimenting with successful and promising prevention strategies, utilized in other countries, and evaluating the results.

## Abbreviations

AUDIT-C	Alcohol Use Disorders Identification Test-Consumption
HCAS	Harvard School of Public Health College Alcohol Study
KCDC	Korean lefts for Disease Control and Prevention
KEDI	Korean Educational Development Institute
KNHANES	Korea National Health and Nutrition Examination Survey
KYRBS	Korea Youth Risk Behavior Web-based Survey
NIH	National Institute of Health
WHO	World Health Organization
